# Discrepant glomerular filtration rate trends from creatinine and cystatin C in patients with chronic kidney disease: results from the KNOW-CKD cohort

**DOI:** 10.1186/s12882-020-01932-4

**Published:** 2020-07-16

**Authors:** Eunjeong Kang, Seung Seok Han, Jayoun Kim, Sue Kyung Park, Wookyung Chung, Yun Kyu Oh, Dong-Wan Chae, Yong-Soo Kim, Curie Ahn, Kook-Hwan Oh

**Affiliations:** 1 Department of Internal Medicine, Ewha Womans University Seoul Hospital, Ewha Womans University College of Medicine, Seoul, South Korea; 2 Department of Internal Medicine, Seoul National University Hospital, Seoul National University College of Medicine, Seoul, South Korea; 3 Medical Research Collaborating Center, Seoul National University College of Medicine, Seoul, South Korea; 4 Department of Preventive Medicine, Seoul National University College of Medicine, Seoul, South Korea; 5 Department of Internal Medicine, Gachon University, Gil Medical Center, Incheon, South Korea; 6 Department of Internal Medicine, Seoul National University Boramae Medical Center, Seoul, South Korea; 7 Department of Internal Medicine, Seoul National University Bundang Hospital, Seongnam, South Korea; 8 Department of Internal Medicine, The Catholic University of Korea, Seoul St. Mary’s Hospital, Seoul, South Korea

**Keywords:** Chronic kidney disease, Creatinine, Cystatin C, Estimated glomerular filtration rate, Trajectory pattern

## Abstract

**Background:**

Serum creatinine (Cr) and cystatin C (CysC) can both be used to estimate glomerular filtration rate (eGFR_Cr_ and eGFR_CysC_). However, certain conditions may cause discrepancies between eGFR trends from Cr and CysC, and these remain undetermined in patients with chronic kidney disease (CKD).

**Methods:**

A total of 1069 patients from the Korean CKD cohort (KNOW-CKD), which enrolls pre-dialytic CKD patients, whose Cr and CysC had been followed for more than 4 years were included in the sample. We performed trajectory analysis using latent class mixed modeling and identified members of the discrepancy group when patient trends between eGFR_Cr_ and eGFR_CysC_ differed. Multivariate logistic analyses with Firth’s penalized likelihood regression models were performed to identify conditions related to the discrepancy.

**Results:**

Trajectory patterns of eGFR_Cr_ were classified into three groups: two groups with stable eGFR_Cr_ (stable with high eGFR_Cr_ and stable with low eGFR_Cr_) and one group with decreasing eGFR_Cr_. Trajectory analysis of eGFR_CysC_ also showed similar patterns, comprising two groups with stable eGFR_CysC_ and one group with decreasing eGFR_CysC_. Patients in the discrepancy group (decreasing eGFR_Cr_ but stable & low eGFR_CysC_; *n* = 55) were younger and had greater proteinuria values than the agreement group (stable & low eGFR_Cr_ and eGFR_CysC_; *n* = 706), differences that remained consistent irrespective of the measurement period (4 or 5 years).

**Conclusions:**

In the present study, we identify conditions related to discrepant trends of eGFR_Cr_ and eGFR_CysC_. Clinicians should remain aware of such potential discrepancies when tracing both Cr and CysC.

## Background

Accurate measurements of glomerular filtration rate (GFR) are important in nephrology. Because actual GFR is difficult to measure and expensive when used for screening, GFR is often estimated using serum creatinine (eGFR_Cr_). However, serum creatinine (Cr) is affected by non-GFR determinants such as muscle mass, body size, diet, and nutritional status [1]. Recently, cystatin C (CysC), which is a 13.3 kDa protein serine protease inhibitor produced by all nucleated cells, was proposed as a marker for estimating GFR [2, 3]. Because CysC is less influenced by muscle mass than other measures, eGFR with CysC (eGFR_CysC_) may reflect GFR more accurately than eGFR with Cr (eGFR_Cr_) in patients with muscle wasting, chronic disease, and limb amputation [1]. The Kidney Disease Improving Global Outcomes guidelines for the evaluation of chronic kidney disease (CKD) recommends using eGFR_Cr_ as an initial assessment of renal function, and eGFR_CysC_ as a confirmation of CKD in certain circumstances when eGFR_Cr_ is less accurate, with an evidence level of 2B. eGFR_CysC_ may be also used in adult patients with eGFR_Cr_ of 45–59 ml/min/1.73 m^2^ who do not have markers of kidney damage, with an evidence level of 2C [4]. Nevertheless, the utility of eGFR_CysC_ and conditions under which eGFR_CysC_ differs from eGFR_Cr_ are unknown.

Intra-individual dynamic change in laboratory measurements provides better prognostic information than cross-sectional data alone [5]. In this respect, trajectory analysis has been applied to evaluate clinical parameters such as blood pressure [6], disability and functional decline [7, 8], and body mass index [9]. Variability in renal function is commonly observed in clinical settings [5]. Previously, trajectory analysis of eGFR demonstrated that CKD patients with catastrophic declining patterns had high rates of co-morbidities and mortality [10, 11].. However, the trajectory patterns of eGFR_CysC_ have not been evaluated. The KNOW-CKD (KoreaN cohort Study for Outcomes in patients With Chronic Kidney Disease), a representative Korean CKD cohort, had traced values of eGFR_CysC_, and we identified certain patients had a discrepancy trend between eGFR_Cr_ and eGFR_CysC_. To identify conditions related to discrepancies, we traced the patterns of both types of eGFR results. To enhance accuracy, both Cr and CysC were measured using calibrations traceable to the international standard reference material.

## Methods

### Study population

**Fig. 1:**
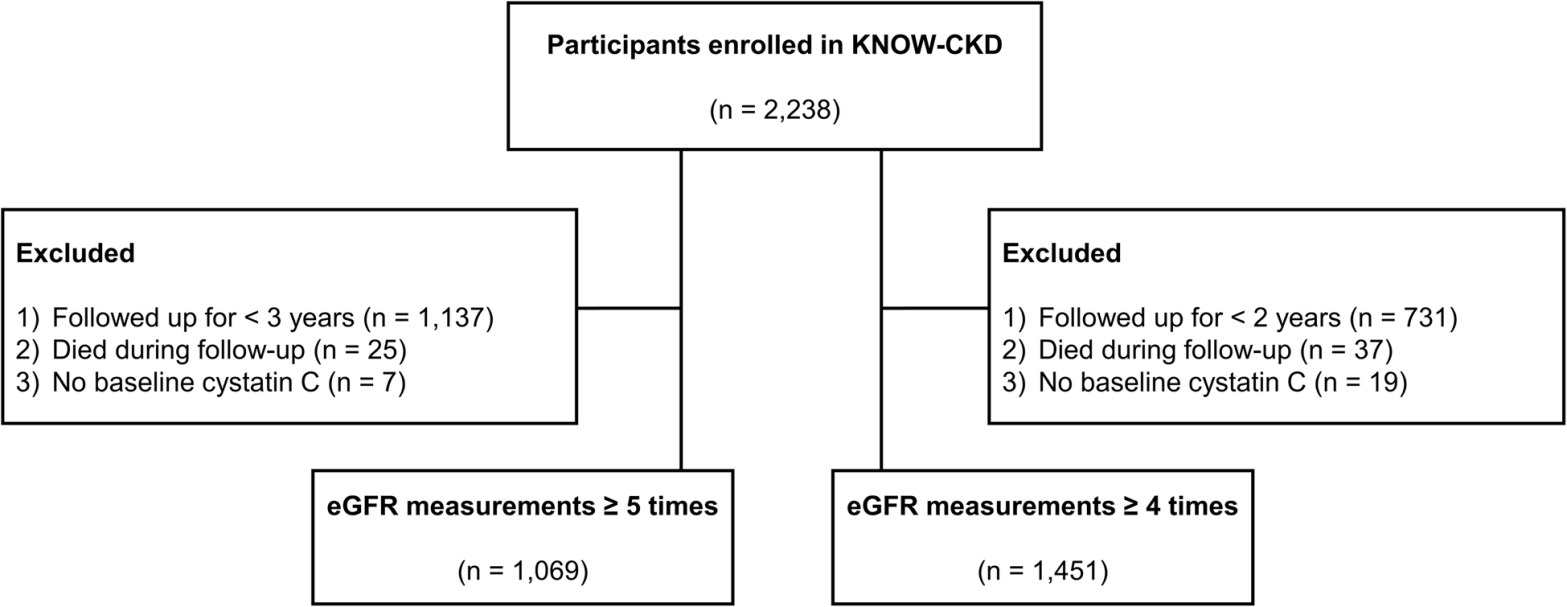
Flow diagram of the study. eGFR, estimated glomerular filtration rate; Cr, creatinine; CysC, cystatin C

Study subjects were selected among participants in the KNOW-CKD, which is a representative prospective Korean pre-dialytic CKD cohort that began enrolling patients in 2011, wherein kidney transplant recipients were not included. The detailed design and method of the KNOW-CKD were described previously [12]. Briefly, a total of 2238 participants were enrolled in the KNOW-CKD study. Both serum Cr and CysC were measured at baseline, 6 months and 1 year after enrollment, and thereafter once per year. Patients who measured both eGFR_Cr_ and eGFR_CysC_ ≥ 5 times from baseline were included. Patients who died during the follow-up period (*n* = 25) and those without baseline CysC (*n* = 7) were excluded. Consequently, 1069 patients were analyzed in the present study. For sensitivity analysis, we defined another group that included patients for whom clinicians measured both eGFR_Cr_ and eGFR_CysC_ ≥ 4 times (Fig. 1).

### Variable measurements

Data for all of the covariates were collected at the time of enrollment including age, sex, comorbidities (diabetes, hypertension, age-adjusted Charlson comorbidity index), body mass index, body surface area, waist and hip circumference, systolic and diastolic pressures, and laboratory findings including white blood cell count, hemoglobin, platelet count, blood urea nitrogen, uric acid, calcium, phosphorus, alkaline phosphatase, total bilirubin, total cholesterol, low density lipoprotein, high density lipoprotein, triglyceride, fasting glucose, albumin, spot urine protein/creatinine ratio (uPCR), and spot urine albumin/creatinine ratio (uACR).

Blood and random voided urine (if possible, second urine in the morning) were collected. All of the samples were measured at a central laboratory (Lab Genomics, Gyeonggi-do, South Korea). Serum Cr was measured by the Jaffe rate blank method using alkaline picrate in a central laboratory and an assay traceable to isotope dilution mass spectrometry (IDMS) (ADVIA® Chemistry Creatinine 2, Siemens, Germany). Serum CysC was measured with a latex-particle enhanced immunoturbidimetric assay (ADVIA® Chemistry Cystatin C Reagents, Siemens, Germany) with calibration traceable to international reference material [13, 14]. The eGFR was estimated by serum Cr or/and CysC using the CKD Epidemiology Collaboration (CKD-EPI) equation [15]. Because of ethical issues and data protection regulations, data that support the findings of the present study cannot be made publicly available.

### Statistical analysis

All statistical analyses were carried out using R (version 3.5.2; The R Foundation for Statistical Computing, Vienna, Austria). Continuous and categorical variables were presented as means±standard deviation and proportions, respectively. We used one-way analysis of variance and the χ^2^ test for comparisons of continuous variables and categorical variables, respectively. For trajectory analysis, we applied latent class mixed modeling (lcmm R package) and the R code is provided in the Supplemental materials. We calculated the entropy, Akaike’s information criteria and Bayesian information criteria for goodness-of-fit statistics and these were described in the Supplemental materials. Subsequently, we defined the discrepancy group as having decreasing eGFR_Cr_ but stable eGFR_CysC_ and the agreement group as having both stable eGFR_Cr_ and eGFR_CysC_.

To identify factors related to discrepant trends, Firth’s penalized likelihood ratio method was used to account for rare events because of potential bias to the maximum likelihood estimator [16–18]. To identify independent conditions related to discrepant trends, univariate and multivariate logistic regression models with backward elimination method were applied. Adjusted variables in multivariate analysis conducted with or without the stepwise conditional method included age, sex, eGFR calculated with Cr and CysC (eGFR_CrCysC_) that represented a renal function, and variables that had *P*-values < 0.1 in univariate analysis. Statistical significance was set as *P* < 0.05 using two-tailed tests.

### Ethics statement

The study protocol was approved by the Institutional Review Board at each participating clinical center [Seoul National University Hospital (1104–089-359), Seoul National University Bundang Hospital (B-1106/129–008), Yonsei University Severance Hospital (4–2011-0163), Kangbuk Samsung Medical Center (2011–01-076), Seoul St. Mary’s Hospital (KC11OIMI0441), Gil Hospital (GIRBA2553), Eulji General Hospital (201105–01), Chonnam National University Hospital (CNUH-2011-092), and Busan Paik Hospital (11–091)]. Written informed consent was obtained from each patient. The study was conducted in accordance with the principles of the Declaration of Helsinki.

## Results

### Baseline characteristics

Baseline characteristics of total enrolled participants were described in Table 1. The mean age of these patients was 53.2 ± 12.1 years and 646 (60.4%) were male. Patients with diabetes and hypertension comprised 283 (26.5%) and 1031 (96.4%) patients, respectively. Mean values for eGFR_Cr_, eGFR_CysC_, and eGFR_CrCysC_ were 58.5 ± 28.9 mL/min/1.73 m^2^, 58.4 ± 31.2 mL/min/1.73 m^2^, and 58.0 ± 30.6 mL/ min/1.73 m^2^, respectively. Median values for uPCR and uACR were 0.4 g/g (0.1–1.0 g/g) and 272.4 mg/g (49.7–705.1 mg/g), respectively. The numbers of patients with uACR 3000 mg/g and uPCR > 3 g/g were 30 (2.8%) and 60 (5.6%), respectively.

**Table 1:** Baseline characteristics of study participants

Variables	Total (*n* = 1069)
Age (years)	53.2 ± 12.1
Male (%)	60.4
Age-adjusted Charlson comorbidity index (%)	3.9 ± 1.8
Low (≤3)	58.7
Moderate (4–5)	27.2
High (6–7)	12.3
Very high (≥8)	1.8
Diabetes mellitus (%)	26.5
Hypertension (%)	96.4
Systolic blood pressure (mmHg)	126.1 ± 14.6
Diastolic blood pressure (mmHg)	76.4 ± 10.3
Body mass index (kg/m^2^)	24.5 ± 3.4
Body surface area (m^2^)	1.7 ± 0.2
Systolic blood pressure (mmHg)	126.1 ± 14.0
Diastolic blood pressure (mmHg)	76.4 ± 10.3
Cause of chronic kidney disease (%)	
Diabetic nephropathy	15.4
Non-diabetic nephropathy	84.6
eGFR (ml/min/1.73 m^2^)	
eGFR_Cr_	58.5 ± 28.9
eGFR_CysC_	58.4 ± 31.2
eGFR_CrCysC_	58.0 ± 30.6
Laboratory findings	
Hemoglobin (g/dL)	13.21 ± 1.85
Blood urea nitrogen (mg/dL)	
Uric acid (mg/dL)	6.90 ± 1.86
Phosphorus (mg/dL)	
Total bilirubin (mg/dL)	
Albumin (g/dL)	4.26 ± 0.35
uPCR (mean, interquartile range)	0.4 (0.1–1.0)
< 0.3 g/g (%)	45.0
0.3–0.9 g/g (%)	30.6
1.0–3.0 g/g (%)	18.8
≥ 3 g/g (%)	5.6
uACR (mean, interquartile range)	272.4 (49.7–705.1)
< 30 mg/g (%)	19.2
30–299 mg/g (%)	33.4
≥ 300 mg/g (%)	47.4
ESRD event (%)	69.0

*eGFR* Estimated glomerular filtration rate; *Cr* Creatinine; *CysC* Cystatin C; *uPCR* Urine protein/creatinine ratio; *uACR* Urine albumin creatinine ratio

### Trajectory patterns of eGFR_Cr_ and eGFR_CysC_

**Fig. 2:**
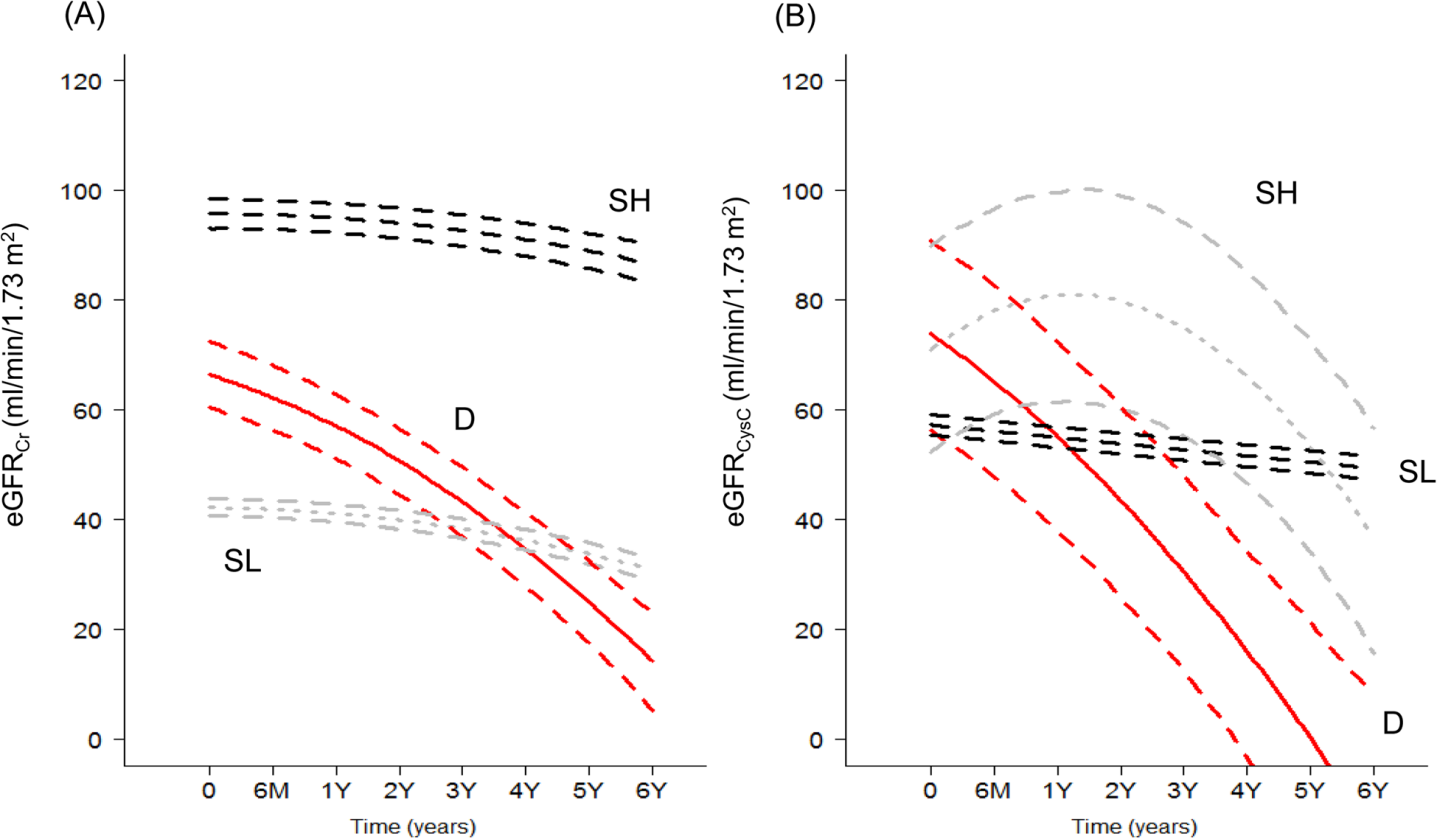
Trajectory patterns of eGFR_Cr_ and eGFR_CysC._ eGFR, estimated glomerular filtration rate; Cr, creatinine; CysC, cystatin C; SH, stable and high eGFR group; SL, stable and low eGFR group; D, decreasing eGFR group

**Fig. 3:**
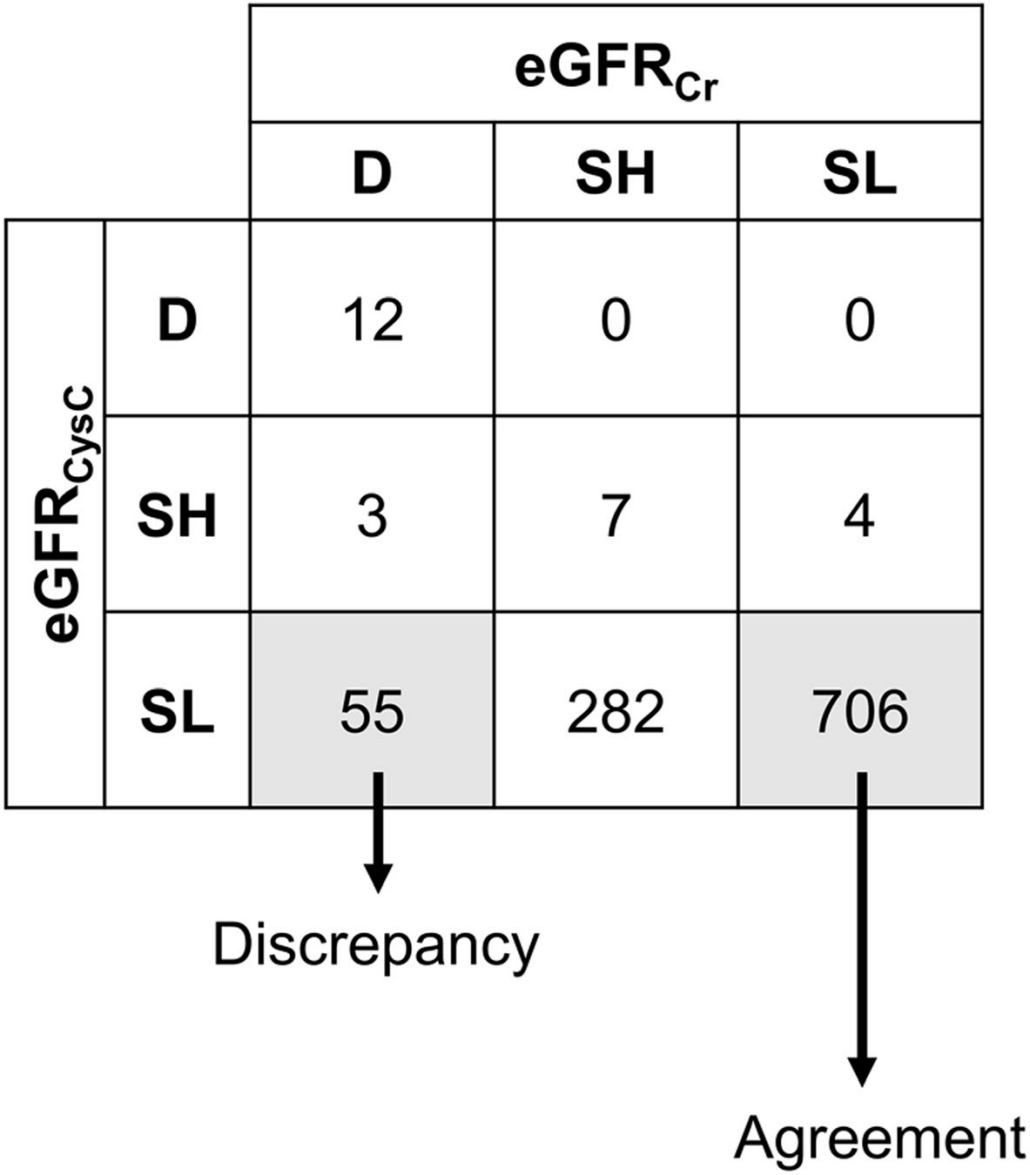
Cross-table between trends of eGFR_Cr_ and eGFR_CysC._ eGFR, estimated glomerular filtration rate; Cr, creatinine; CysC, cystatin C; SH, stable and high eGFR group; SL, stable and low eGFR group; D, decreasing eGFR group

The relationship between baseline eGFR_Cr_ and eGFR_CysC_ is shown as a Bland-Altman plot (Supplemental Figure 1). The mean value of difference was 0.148, and standard deviation was 11.327. The correlation coefficient (r) between eGFR_Cr_ and eGFR_CysC_ was 0.93. We identified three distinct trajectory patterns for eGFR_Cr_ (Fig. 2): two groups with stable eGFR_Cr_ (stable with high eGFR_Cr_ [SH] and stable with low eGFR_Cr_ [SL]) and one group with decreasing eGFR_Cr_ (D). Trajectories of eGFR_CysC_ also showed similar patterns, with two groups with stable eGFR_CysC_ (SH and SL) and one group with decreasing eGFR_CysC_ (D). Baseline characteristics according to the group of eGFR_Cr_ and eGFR_CysC_ were described in Supplemental Table 1. Particularly, 69% of the ESRD events were occurred in the decreasing eGFRCr (D) group. We conducted cross-tabulation using these groups (Fig. 3). Most patients (97.6%; *n* = 1043) were classified into the SL group in eGFR_CysC_, followed by the SH (1.31%, *n* = 14) and D (1.12%, *n* = 12) groups. There were small numbers of patients in the D and SH groups with eGFR_CysC_.

### Conditions related to discrepant trends between eGFR_Cr_ and eGFR_CysC_

Table 2 summarizes baseline characteristics according to discrepant trends. The patients in the agreement group were older than those in the discrepancy group. There were no differences in underlying disease, including diabetes and hypertension, or body mass index. Body surface area was greater in the discrepancy group than in the agreement group. Proteinuria values represented by uPCR and uACR and baseline renal function evaluated by eGFR_Cr_, eGFR_CysC_, and eGFR_CrCysC_ were higher in the discrepancy group than in the agreement group.

**Table 2:** Baseline characteristics according to discrepancy between the trends of eGFR_Cr_ and eGFR_CysC_

	Discrepancy (*n* = 55)	Agreement (*n* = 706)	*P*
Age (years)	44.8 ± 10.3	56.8 ± 10.7	< 0.001
Male (%)	61.8	62.2	0.957
Age-adjusted Charlson comorbidity index (%)	2.4 ± 1.7	4.0 ± 1.7	< 0.001
Low (≤3)	74.5	42.9	
Moderate (4–5)	21.8	36.5	
High (6–7)	3.6	18.1	
Very high (≥8)	0	2.4	
Diabetes (%)	27.3	31.4	0.622
Hypertension (%)	98.2	98.9	1.000
Systolic blood pressure (mmHg)	128.0 ± 13.0	125.9 ± 14.7	0.306
Diastolic blood pressure (mmHg)	78.4 ± 10.7	75.7 ± 10.1	0.062
Body mass index (kg/m^2^)	24.4 ± 4.0	24.7 ± 3.3	0.514
Body surface area (m^2^)	1.8 ± 0.2	1.7 ± 0.2	0.036
Systolic blood pressure (mmHg)	128.0 ± 13.0	125.9 ± 14.7	0.306
Diastolic blood pressure (mmHg)	78.4 ± 10.7	75.7 ± 10.1	0.062
Cause of chronic kidney disease (%)			0.924
Non-diabetic nephropathy	81.8	80.3	
Diabetic nephropathy	18.2	19.7	
eGFR (ml/min/1.73 m^2^)			
eGFR_Cr_	66.4 ± 16.6	41.9 ± 15.2	< 0.001
eGFR_CysC_	58.2 ± 19.72	41.6 ± 17.9	< 0.001
eGFR_CrCysC_	61.0 ± 18.3	40.8 ± 16.1	< 0.001
Laboratory findings			
Hemoglobin (g/dL)	13.4 ± 1.7	12.9 ± 1.9	0.029
Blood urea nitrogen (mg/dL)	20.8 ± 5.8	20.2 ± 8.6	< 0.001
Uric acid (mg/dL)	6.8 ± 1.7	7.4 ± 1.7	0.020
Phosphorus (mg/dL)	3.5 ± 0.5	3.6 ± 0.6	0.041
Total bilirubin (mg/dL)	0.7 ± 0.3	0.7 ± 0.3	0.087
Albumin (g/dL)	4.2 ± 0.3	4.2 ± 0.3	0.967
uPCR (mean, interquartile range)	0.5 (0.2–1.5)	0.4 (0.1–1.0)	0.054
< 0.3 g/g (%)	30.9	41.3	0.011
0.3–0.9 g/g (%)	27.3	33.1	
1.0–3.0 g/g (%)	27.3	20.6	
≥ 3.0 g/g (%)	14.5	5.0	
uACR (mean, interquartile range g)	427 (120–1206)	295 (72–744)	0.040
< 30 mg/g (%)	7.3	14.5	0.202
30–299 mg/g (%)	32.7	36.1	
≥ 300 mg/g (%)	60.0	49.4	

*eGFR* Estimated glomerular filtration rate; *Cr* Creatinine; *CysC* Cystatin C; *uPCR* Urine protein/creatinine ratio; *uACR* Urine albumin creatinine ratio

When the discrepancy group was set as the dependent variable, younger age and proteinuria were selected as predictors of discrepancies between trends of eGFR_Cr_ and eGFR_CysC_. When the backward elimination method was applied (model 2 in Table 3), age and proteinuria remained significant for predicting the discrepancy of trends. These results remained consistent in the subgroup analyses according to the age and proteinuria (Supplementary Tables 4, 5).

**Table 3:** Analysis to identify conditions related to discrepant trends of eGFR_Cr_ and eGFR_CysC_

	Model 1		Model 2	
Variables	OR (95% CI)	*P*	OR (95% CI)	*P*
Age	0.92 (0.89–0.95)	< 0.001	0.92 (0.89–0.95)	< 0.001
Male	1.60 (0.61–4.26)	0.343		
Age-adjusted CCI				
Low (≤3)	Reference			
Moderate (4–5)	1.30 (0.53–3.12)	0.563		
High (6–7)	1.66 (0.28–7.28)	0.541		
Very high (≥8)	2.48 (0.02–30.54)	0.608		
Body surface area	2.01 (0.18–20.83)	0.562		
Diastolic blood pressure	1.01 (0.98–1.04)	0.579		
Hemoglobin	0.90 (0.71–1.13)	0.350		
Blood urea nitrogen	0.99 (0.92–1.05)	0.661		
Uric acid	0.91 (0.74–1.12)	0.379		
Phosphorus	0.83 (0.42–1.62)	0.590		
Total bilirubin	2.00 (0.52–7.35)	0.307		
uPCR (g/g)				
< 0.3	Reference		Reference	
0.3–0.9	1.69 (0.49–5.26)	0.392	1.53 (0.68–3.45)	0.305
1.0–3.0	4.54 (0.95–21.44)	0.058	3.32 (1.43–7.84)	0.006
≥ 3.0	17.87 (3.14–102.19)	0.001	12.38 (4.07–37.39)	< 0.001
uACR (mg/g)				
< 30	Reference			
30–299	2.53 (0.74–10.73)	0.145		
≥ 300	1.55 (0.27–10.19)	0.630		
eGFR_CrCysC_	1.07 (1.04–1.10)	< 0.001	1.07 (1.05–1.09)	< 0.001

Model 1: Adjusted for age, sex, eGFR_CrCys_ and the variables which had *P* value less than 0.1 in univariate analysis

Model 2: Model 1 with backward elimination method

*CCI* Charlson comorbidities index; *OR* Odds ratio; *CI* Confidence interval; *uPCR* Urine protein/creatinine ratio; *uACR* Urine albumin/creatinine ratio; *eGFR*, estimated glomerular filtration rate; *Cr* Creatinine; *CysC* Cystatin C

### Sensitivity analysis with patients for whom eGFRs were measured ≥4 times

The sensitivity analysis was conducted in patients for whom eGFRs were measured more than 4 times (*n* = 1451). The results for most baseline features were similar to those of the previous patient group (Supplemental Table 6). Their mean age was 53.2 ± 12.1 years old and 59.5% of enrolled patients were male. Diabetic patients accounted for 29.3%. Mean values of eGFR_Cr_, eGFR_CysC_ and eGFR_CrCysC_ were 57.5 ± 29.6 mL/min/1.73 m^2^, 57.1 ± 31.4 mL/min/1.73 m^2^, and 56.9 ± 31.1 mL/ min/1.73 m^2^, respectively.

The trajectory patterns of eGFR_Cr_ and eGFR_CysC_ were classified into 3 groups (Supplemental Figure 1), and there were discrepancies between trends similar to those observed in the main analysis (Supplemental Figure 2). In multivariate analysis, young patient age, proteinuria, and other variables such as male sex and large body surface area had tendencies for discrepancy compared with the counterpart groups (Supplemental Table 7).

## Discussion

Information about eGFRs trends may be more helpful to predict prognosis than single measurements of eGFR. Although CysC has been used as an additional parameter to calculate GFR, eGFR_CysC_ trends have not been evaluated and compared to those of eGFR_Cr_. In the present study, we first compared eGFR_Cr_ and eGFR_CysC_ trends and found that certain factors such as young age and proteinuria were related to discrepancies in trends between two eGFRs.

In the present study, we identified young age as a condition related to discrepancies between two eGFR trends, and the possible mechanisms are described as follows. There was a non-linear association between age and CysC concentration [19], and the increment rates of CysC levels were accelerated in patients aged over 50–60 years [20, 21]. Serum Cr remained relatively constant in healthy individuals between 20 and 70 years old [22]. Because there is a gap between the time point of increasing Cr and CysC, age may be a factor underlying discrepancies between eGFR trends. Additionally, when the CKD-EPI equation was developed, a large number of young patients were included from various diabetic cohorts [23], so that the proportions of younger diabetic patients differed from those in more recent studies (≤40 years, 11%; and 41–50 years, 20% in the KNOW-CKD cohort vs. ≤40 years, > 40% in the CKD-EPI-developing cohort). Such baseline differences might affect the non-GFR determinants of CysC because CysC is associated with insulin resistance, obesity, hypertension, and oxidative stress, which in turn are closely dependent on diabetes [24–26]. Inflammation could be a reason for the discrepancy between trends of eGFR_Cr_ and eGFR_CysC_, as inflammation is a representative determinant of CysC [27]. Although a wide ranges of inflammatory markers were not measured in the study cohort, young and old participants might have different inflammatory milieu that affects eGFR_CysC_ trends. Because these hypotheses have not been thoroughly tested, further evaluations regarding the mechanisms underlying this phenomenon are needed.

Most filtered CysC is reabsorbed and metabolized by the proximal tubule cells [28, 29]. Previous study identified that the concentration of CysC was influenced by urine protein excretion, an influence stronger than that of Cr [30]. Similarly, several studies suggest that heavy proteinuria influenced renal handling of CysC [31, 32]. The association between urinary CysC and proteinuria was predominant in pediatric cases with nephrotic syndrome compared with controls [32]. Proteinuria itself decreases the tubular uptake of low molecular weight proteins, including CysC, primarily throughout the competition for a common transport mechanism in the preclinical model [31]. The present findings regarding the relationship between proteinuria and discrepant trends might be attributable to these factors.

Non-GFR determinants are well-known for serum Cr and CysC, respectively. A representative non-GFR determinant for Cr is muscle mass. Body mass index is a simple index for body composition but does not distinguish between excess fat, muscle, and bone mass [33, 34]. In the present study, we did not detect the independent significance of body mass index underlying the discrepancy between eGFR trends, although a dependent relationship with body surface area was detected. This difference might be because body mass index and body surface area do not reflect muscle mass. In the present study, the mean body mass index was 24.5 ± 3.4 kg/m^2^, which was lower than that of another CKD cohort (32.1 ± 7.9 kg/m^2^ in CRIC) [35]. In this respect, population-related factors also hamper the distinctive relationship between body mass index and muscle mass and thus, the effects of body mass index and body surface area might disappear in the final analysis.

The study has some limitations that deserve attention. The number of subjects was modest, although the statistical power was sufficient. Particularly, we could not compare the discrepant trends between some groups with low patient numbers. The study sample was entirely comprised of East Asians and CKD-EPI eGFR equations were not validated in the Korean population. As noted above, non-eGFR_Cr_ determinants such as muscle mass differed from those of individuals of European descent, which warrants further study to identify other significant conditions. The present findings were obtained from patients with non-dialytic CKD, and thus, the application of results to healthy individuals or the general population is limited. Standard measurements of GFR such as inulin excretion rate were not available for the study cohort, and such data would be useful to determine which trend was more accurate.

The results of the present study demonstrate discrepant conditions between trends from eGFR_Cr_ and eGFR_CysC_. Although further studies are needed to confirm our findings in other independent cohorts, clinicians should remain aware that discrepant conditions may occur when both Cr and CysC are used to evaluate and trace renal function. Because the present guidelines do not urge caution when determining the condition of eGFR_CysC_, our results may constitute the basis of future updates.

## Conclusions

In conclusion, we identify conditions related to discrepant trends of eGFR_Cr_ and eGFR_CysC_. Clinicians should remain aware of such potential discrepancies when tracing both Cr and CysC.

## Supplementary information

**Additional file 1.**

## Data Availability

The dataset can be available that is within the perspective of the scientific objectives of KNOW-CKD and researchers who approved by the KNOW-CKD investigators can be accessed the data (http://www.know-ckd.org/ckd/main/main.html).

## Abbreviations

Cr: Creatinine
CysC: Cystatin C
eGFR: Estimated glomerular filtration rate
GFR: Glomerular filtration rate
KNOW-CKD: KoreaN cohort study for outcomes in patients with chronic kidney disease
CKD: Chronic kidney disease
uPCR: Spot urine protein/creatinine ratio
uACR: Spot urine albumin/creatinine ratio
IDMS: Isotope dilution mass spectrometry
CKD-EPI: Chronic kidney disease epidemiology collaboration
SH: Stable with high eGFR
SL: Stable with low eGFR
D: Decreasing eGFR
