# Supplementary material for: Discrepant glomerular filtration rate trends from creatinine and cystatin C in patients with chronic kidney disease: results from the KNOW-CKD cohort
Source: BMC Nephrol. 2020 Jul 16;21:280. doi: 10.1186/s12882-020-01932-4 (PMC7364655; doi:10.1186/s12882-020-01932-4)

**Discrepant glomerular filtration rate trends from creatinine and cystatin C in patients with chronic kidney disease: results from the KNOW-CKD cohort**

**Technical appendix, Supplemental Table S1 to S7 and Supplemental Figure S1 to S3**

**Corresponding author:**

Kook-Hwan Oh (khoh@snu.ac.kr)

**Contents**

**Appendix. Technical appendix for trajectory analysis** ……………………………………. 2

**Supplemental Table 1** …………………………………………………………..………….... 4

**Supplemental Table 2** …………………………………………………………..…………… 6

**Supplemental Table 3** …………..………………………………………………..…………. 8

**Supplemental Table 4** ………….………………………………………………..………… 10

**Supplemental Table 5** ………….………………………………………………………...…11

**Supplemental Table 6** ………..……………………………………………………………..12

**Supplemental Table 7** ………..……………………………………………………………..14

**Supplemental Figure 1** …..…..……………………………………………………..……….16

**Supplemental Figure 2** ……………………………………………………………..……….17

**Supplemental Figure 3** ………..…………………………………………………………….18

**Appendix: Technical appendix for trajectory analysis**

In this appendix, we provide the R code that we used to estimate the final model, and the results for goodness-of-fit statistics.

- *R code for trajectory analysis for eGFR_Cr_ using LCMM*

## data set ##

rm(list=ls())

setwd("")

install.packages(“lcmm”)

install.packages(“reshpae2”)

install.packages(“extrafont”)

require(lcmm)

require(reshape2)

require(extrafont)

dat=read.csv("*file name*",header=T)

dat_cr=dat[,c(1,25,31,37,43,49,55,61,67)]

dat_cysc=dat[,c(1,26,32,38,44,50,56,62,68)]

dat_crcysc=dat[,c(1,27,33,39,45,51,57,63,69)]

### 1. Trajectory analysis for creatinine ###

dat_cr2=melt(dat_cr, id.vars= "SUBJECT_NO",

measure.vars=c("EPI_CR","EPI_CR_M6","EPI_CR_Y1","EPI_CR_Y2","EPI_CR_Y3","EPI_CR_Y4","EPI_CR_Y5","EPI_CR_Y6"),

variable.name="time1",value.name="cr")

levels(dat_cr2$time1)[levels(dat_cr2$time1)=="EPI_CR"]=1

levels(dat_cr2$time1)[levels(dat_cr2$time1)=="EPI_CR_M6"]=2

levels(dat_cr2$time1)[levels(dat_cr2$time1)=="EPI_CR_Y1"]=3

levels(dat_cr2$time1)[levels(dat_cr2$time1)=="EPI_CR_Y2"]=4

levels(dat_cr2$time1)[levels(dat_cr2$time1)=="EPI_CR_Y3"]=5

levels(dat_cr2$time1)[levels(dat_cr2$time1)=="EPI_CR_Y4"]=6

levels(dat_cr2$time1)[levels(dat_cr2$time1)=="EPI_CR_Y5"]=7

levels(dat_cr2$time1)[levels(dat_cr2$time1)=="EPI_CR_Y6"]=8

dat_cr2$cr=as.numeric(dat_cr2$cr)

dat_cr2$time1=as.numeric(dat_cr2$time1)

dat_cr2=na.omit(dat_cr2)

cr_res1=lcmm(cr~time1 + I(time1^2), random=~time1, mixture=~time1+ I(time1^2), subject='SUBJECT_NO', ng=3, data=dat_cr2)

summary(cr_res1)

summarytable(cr_res1,which="entropy")

- Results for Goodness-of-fit statistics

|  | **Entropy** | **AIC** | **BIC** |
| --- | --- | --- | --- |
| eGFR_Cr_ | 0.9127 | 53168.48 | 53282.79 |
| eGFR_CysC_ | 0.9883 | 52540.54 | 52654.95 |
| eGFR_CrCysC_ | 0.8081 | 50527.22 | 50641.63 |

**Supplemental Table 1. Baseline characteristics according to the group of eGFR_Cr_ and eGFR_CysC_**

| eGFR (Cr) | D | D | SH | SL | D | SH | SL |
| --- | --- | --- | --- | --- | --- | --- | --- |
| eGFR (CysC) | D | SH | SH | SH | SL | SL | SL |
| Number of patients | (n=12) | (n=3) | (n=7) | (n=4) | (n=55) | (n=282) | (n=706) |
| Age (years) | 49.8±9.8 | 33.7±5.7 | 42.4±15.3 | 47.2±15.0 | 44.8±10.3 | 46.4±11.8 | 56.8±10.7 |
| Male (%) | 66.7 | 33.3 | 28.6 | 0.0 | 38.2 | 44.0 | 37.8 |
| Age-adjusted Charlson comorbidity index (%) | |  |  |  |  |  |  |
| Low (≤ 3) | 66.7 | 66.7 | 100.0 | 75.0 | 74.5 | 93.6 | 42.9 |
| Moderate (4–5) | 16.7 | 33.3 | 0.0 | 25.0 | 21.8 | 6.0 | 36.5 |
| High (6–7) | 0.0 | 0.0 | 0.0 | 0.0 | 3.6 | 0.4 | 18.1 |
| Very high (≥8) | 16.7 | 0.0 | 0.0 | 0.0 | 0.0 | 0.0 | 2.4 |
| Diabetes mellitus (%) | 41.7 | 33.3 | 14.3 | 0.0 | 27.3 | 13.8 | 31.4 |
| Hypertension (%) | 91.7 | 100.0 | 100.0 | 100.0 | 98.2 | 90.1 | 98.9 |
| Systolic blood pressure (mmHg) | 127.1±18.8 | 126.3±13.9 | 141.4±16.0 | 116.0±14.4 | 128.0±13.0 | 125.9±14.3 | 125.9±14.7 |
| Diastolic blood pressure (mmHg) | 76.9±13.1 | 78.3±5.9 | 86.1±10.6 | 72.0±8.5 | 78.4±10.7 | 77.6±10.6 | 75.7±10.1 |
| Body mass index (kg/m^2^) | 24.8±2.2 | 26.2±1.7 | 26.5±4.3 | 23.5±3.1 | 24.4±4.0 | 24.0±3.6 | 24.7±3.3 |
| Body surface area (m^2^) | 1.7±0.2 | 1.8±0.1 | 1.8±0.2 | 1.9±0.0 | 1.8±0.2 | 1.7±0.2 | 1.7±0.2 |
| Cause of chronic kidney disease (%) |  |  |  |  |  |  |  |
| Diabetic nephropathy | 33.3 | 0.0 | 0.0 | 0.0 | 18.2 | 4.3 | 19.7 |
| Non-diabetic nephropathy | 66.7 | 100.0 | 100.0 | 100.0 | 81.8 | 95.7 | 15.5 |
| eGFR (ml/min/1.73 m^2^) |  |  |  |  |  |  |  |
| eGFR_Cr_ | 71.2±19.4 | 62.6±6.1 | 96.8±20.1 | 56.4±14.4 | 66.4±16.6 | 97.3±16.2 | 41.8±15.1 |
| eGFR_CysC_ | 72.7±21.1 | 72.0±16.2 | 84.9±16.2 | 51.1±10.4 | 58.2±19.7 | 99.1±19.8 | 41.6±17.9 |
| eGFR_CrCysC_ | 70.6±16.6 | 65.9±10.7 | 89.8±13.8 | 52.7±11.2 | 61.0±18.3 | 99.3±18.5 | 40.8±16.1 |
| Laboratory findings |  |  |  |  |  |  |  |
| Hemoglobin (g/dL) | 12.7±1.6 | 13.6±1.5 | 13.9±1.8 | 14.8±1.7 | 13.4±1.7 | 14.0±1.6 | 12.9±1.9 |
| Blood urea nitrogen (mg/dL) | 21.0±8.3 | 17.2±5.3 | 18.3±11.2 | 23.6±7.0 | 20.8±5.8 | 14.4±4.0 | 28.5±11.7 |
| Uric acid (mg/dL) | 6.6±2.0 | 7.8±2.6 | 6.2±2.2 | 8.4±1.3 | 6.8±1.7 | 5.8±1.7 | 7.4±1.7 |
| Phosphorus (mg/dL) | 3.7±0.6 | 3.9±0.4 | 3.3±0.8 | 3.5±0.2 | 3.5±0.5 | 3.5±0.6 | 3.6±0.6 |
| Total bilirubin (mg/dL) | 0.7±0.2 | 1.1±0.2 | 0.6±0.2 | 0.6±0.3 | 0.7±0.3 | 0.8±0.4 | 0.7±0.3 |
| Albumin (g/dL) | 3.9±0.4 | 4.5±0.2 | 4.1±0.3 | 4.1±0.4 | 4.2±0.3 | 4.3±0.4 | 4.2±0.3 |
| Urine protein/creatinine ratio (%) |  |  |  |  |  |  |  |
| <0.3 (g/g) | 16.7 | 0.0 | 14.3 | 0.0 | 30.9 | 60.0 | 41.3 |
| 0.3–0.9 (g/g) | 16.7 | 66.7 | 57.1 | 25.0 | 27.3 | 24.6 | 33.1 |
| 1.0–3.0 (g/g) | 33.3 | 33.3 | 14.3 | 75.0 | 27.3 | 11.1 | 20.6 |
| ≥3 (g/g) | 33.3 | 0.0 | 14.3 | 0.0 | 14.5 | 4.3 | 5.0 |
| Urine albumin/creatinine ratio (%) |  |  |  |  |  |  |  |
| <30 (mg/g) | 8.3 | 0.0 | 0.0 | 0.0 | 7.3 | 34.6 | 14.5 |
| 30–299 (mg/g) | 8.3 | 0.0 | 28.6 | 0.0 | 32.7 | 28.9 | 36.1 |
| ≥300 (mg/g) | 83.3 | 100.0 | 71.4 | 100.0 | 60.0 | 36.4 | 49.4 |
| ESRD event (%) | 41.7 | 0.0 | 0.0 | 0.0 | 27.3 | 0.0 | 16.6 |

D, decreasing eGFR group; SH, stable and high eGFR group; SL, stable and low eGFR group; eGFR, estimated glomerular filtration rate; Cr, creatinine; CysC, cystatin C; ESRD, end-stage renal disease

**Supplemental Table 2. Other baseline characteristics according to the discrepancy between trends of eGFR_Cr_ and eGFR_CysC_**

| Variables | Discrepancy  (n=55) | Agreement  (n=706) | *P* |
| --- | --- | --- | --- |
| Comorbidities (%) |  |  |  |
| Myocardial infarction | 0 | 1.1 | 0.915 |
| Congestive heart failure | 0 | 1.4 | 0.784 |
| Peripheral vascular disease | 1.8 | 3.7 | 0.733 |
| Cerebrovascular disease | 0 | 6.4 | 0.102 |
| Dementia | 0 | 0 |  |
| Chronic obstructive pulmonary disease | 0 | 0.4 | 1.000 |
| Connective tissue disease | 1.8 | 7.2 | 0.210 |
| Peptic ulcer disease | 0 | 2.7 | 0.433 |
| Liver disease, mild | 1.8 | 1.6 | 1.000 |
| Hemiplegia | 0 | 0.4 | 1.000 |
| Renal disease, moderate or severe | 69.1 | 94.1 | <0.001 |
| Diabetes with end organ damage | 23.6 | 26.9 | 0.711 |
| Any malignancy | 0 | 0 |  |
| Lymphoma | 0 | 0 |  |
| Leukemia | 0 | 0 |  |
| Liver disease, moderate to severe | 0 | 0 |  |
| Metastatic solid malignancy | 0 | 0 |  |
| Acquired immunodeficiency syndrome | 0 | 0 |  |
| Waist (cm) | 88.5±10.7 | 87.7±9.5 | 0.566 |
| Hip (cm) | 97.2±6.8 | 96.8±6.7 | 0.662 |
| Waist hip ratio | 0.9±0.1 | 0.9±0.1 | 0.749 |
| Blood findings |  |  |  |
| White blood cell count (×10^3^/uL) | 7.0±1.8 | 6.7±1.9 | 0.136 |
| Platelet count (×10^3^/uL) | 229.8±59.4 | 216.7±58.7 | 0.116 |
| Calcium (mg/dL) | 9.3±0.3 | 9.2±0.5 | 0.065 |
| Alkaline phosphatase (IU/L) | 73.2±46.7 | 85.2±58.1 | 0.076 |
| Total cholesterol (mg/dL) | 177.8±31.7 | 169.1±36.0 | 0.081 |
| Low density lipoprotein (mg/dL) | 96.9±30.6 | 92.0±29.2 | 0.235 |
| High density lipoprotein (mg/dL) | 51.8±17.6 | 48.1±14.8 | 0.084 |
| Triglyceride (mg/dL) | 171.8±88.6 | 161.7±100.9 | 0.477 |
| Fasting blood sugar (mg/dL) | 112.4±48.5 | 109.3±36.8 | 0.643 |
| Ejection fraction (%) |  |  | 1.000 |
| <45 | 0 | 0.4 |  |
| ≥45 | 100 | 99.6 |  |

**Supplemental Table 3. Univariate logistic regression analysis for conditions related to discrepancy between the trends of eGFR_Cr_ and eGFR_CysC_**

|  | OR (95% CI) | *P* |
| --- | --- | --- |
| Age | 0.91 (0.89–0.93) | <0.001 |
| Male | 1.02 (0.58–1.78) | 0.934 |
| Underlying disease |  |  |
| Diabetes | 0.83 (0.44–1.50) | 0.551 |
| Hypertension | 0.44 (0.10–4.20) | 0.406 |
| Peripheral vascular disease | 0.71 (0.08–2.79) | 0.668 |
| Connective tissue disease | 0.30 (0.04–1.34) | 0.142 |
| Liver disease, mild | 1.66 (0.19–7.18) | 0.586 |
| Renal disease, moderate or severe | 0.14 (0.07–0.27) | <0.001 |
| Diabetes with end organ damage | 0.86 (0.44–1.58) | 0.641 |
| Age-adjusted Charlson comorbidity index |  |  |
| Low (≤ 3) | Reference |  |
| Moderate (4–5) | 0.35 (0.18–0.66) | <0.001 |
| High (6–7) | 0.14 (0.03–0.43) | <0.001 |
| Very high (≥8) | 0.20 (0.00–1.59) | 0.161 |
| Body mass index | 0.97 (0.89–1.05) | 0.455 |
| Body surface area | 5.49 (1.11–27.00) | 0.036 |
| Waist | 1.01 (0.98–1.04) | 0.567 |
| Hip | 1.01 (0.97–1.05) | 0.646 |
| Waist hip ratio | 1.93 (0.03–152.04) | 0.762 |
| Systolic blood pressure | 1.01 (0.99–1.03) | 0.293 |
| Diastolic blood pressure | 1.03 (0.99–1.05) | 0.063 |
| Cause of chronic kidney disease |  |  |
| Non–diabetic nephropathy | Reference |  |
| Diabetic nephropathy | 0.94 (0.44–1.81) | 0.858 |
| Serum Lab Findings |  |  |
| White blood cell count | 1.00 (1.00–1.00) | 0.133 |
| Platelet count | 1.00 (1.00–1.01) | 0.114 |
| Hemoglobin | 1.18 (1.02–1.37) | 0.030 |
| Blood urea nitrogen | 0.89 (0.85–0.93) | <0.001 |
| Uric acid | 0.83 (0.70–0.97) | 0.020 |
| Albumin | 0.99 (0.45–2.35) | 0.990 |
| Calcium | 1.58 (0.85–2.94) | 0.148 |
| Phosphorus | 0.60 (0.36–0.98) | 0.040 |
| Alkaline phosphatase | 1.00 (0.99–1.00) | 0.147 |
| Total bilirubin | 2.66 (1.03–6.43) | 0.043 |
| Total cholesterol | 1.01 (0.99–1.01) | 0.081 |
| Low density lipoprotein | 1.01 (0.99–1.01) | 0.225 |
| High density lipoprotein | 1.01 (1.00–1.03) | 0.084 |
| Triglyceride | 1.00 (0.99–1.00) | 0.411 |
| Fasting blood sugar | 1.00 (1.00–1.01) | 0.429 |
| C–reactive protein | 0.87 (0.68–1.01) | 0.110 |
| Urine protein/creatinine ratio (g/g) |  |  |
| <0.3 | Reference |  |
| 0.3–0.9 | 1.11 (0.54–2.24) | 0.780 |
| 1.0–3.0 | 1.77 (0.86–3.62) | 0.118 |
| ≥3 | 3.99 (1.57–9.51) | 0.005 |
| Urine albumin/creatinine ratio (mg/g) |  |  |
| <30 | Reference |  |
| 30–299 | 1.66 (0.62–5.42) | 0.328 |
| ≥300 | 2.19 (0.88–6.93) | 0.097 |
| Ejection fraction | 1.00 (0.96–1.05) | 0.949 |
| Renal function |  |  |
| eGFR_Cr_ | 1.10 (1.08–1.13) | <0.001 |
| eGFR_CysC_ | 1.04 (1.03–1.06) | <0.001 |
| eGFR_CrCysC_ | 1.07 (1.05–1.09) | <0.001 |

OR, odds ratio; CI, confidence interval; eGFR, estimated glomerular filtration rate; Cr, creatinine; CysC, cystatin C

**Supplemental Table 4. Subgroup analysis according to urine microalbumin-to-creatinine ratio to identify conditions related to discrepant trends of eGFR_Cr_ and eGFR_CysC_**

| **Variables** | **uACR <300 mg/g** | | | | **uACR ≥300 mg/g** | | | |
| --- | --- | --- | --- | --- | --- | --- | --- | --- |
|  | **Model 1** | | **Model 2** | | **Model 1** | | **Model 2** | |
|  | **OR (95% CI)** | ***P*** | **OR (95% CI)** | ***P*** | **OR (95% CI)** | ***P*** | **OR (95% CI)** | ***P*** |
| Age | 0.87 (0.81-0.93) | 0.000 | 0.88 (0.83-0.93) | <0.001 | 0.94 (0.90-0.99) | 0.008 | 0.95 (0.91-0.98) | 0.003 |
| Male | 1.88 (0.36-1.01) | 0.450 |  |  | 1.61 (0.50-5.27) | 0.426 |  |  |
| Age-adjusted CCI |  |  |  |  |  |  |  |  |
| Low (≤3) | Reference |  |  |  | Reference |  |  |  |
| Moderate (4–5) | 1.80 (0.29-8.78) | 0.492 |  |  | 1.45 (0.53-3.94) | 0.461 |  |  |
| High (6–7) | 1.99 (0.01-29.42) | 0.692 |  |  | 1.65 (0.25-8.77) | 0.579 |  |  |
| Very high (≥8) | 17.32 (0.11-34.69) | 0.191 |  |  | 7.12 (0.05-132.05) | 0.331 |  |  |
| Body surface area | 0.58 (0.01-29.55) | 0.788 |  |  | 6.54 (0.29-146.94) | 0.236 |  |  |
| Diastolic blood pressure | 1.01 (0.95-1.06) | 0.840 |  |  | 1.00 (0.96-1.04) | 0.989 |  |  |
| Hemoglobin | 0.91 (0.56-1.45) | 0.693 |  |  | 0.93 (0.70-1.23) | 0.617 |  |  |
| Blood urea nitrogen | 1.03 (0.90-11.04) | 0.613 |  |  | 0.95 (0.90-1.05) | 0.517 |  |  |
| Uric acid | 0.79 (0.56-1.10) | 0.166 |  |  | 1.01 (0.76-1.32) | 0.958 |  |  |
| Phosphorus | 0.74 (0.22-2.45) | 0.632 |  |  | 0.87 (0.37-1.97) | 0.733 |  |  |
| Total bilirubin | 4.82 (0.68-3.02) | 0.114 |  |  | 0.51 (0.08-2.77) | 0.447 |  |  |
| eGFR_CrCysC_ | 1.07 (1.02-1.11) | 0.002 | 1.07 (1.04-1.10) | <0.001 | 1.05 (1.02-1.10) | 0.001 | 1.06 (1.04-1.09) | <0.001 |

Model 1: Adjusted for age, sex, age-adjusted CCI, body surface area, diastolic blood pressure, hemoglobin, blood urea nitrogen, uric acid, phosphorus, total bilirubin and eGFR_CrCys_.

Model 2: Model 1 with backward elimination method.

CCI, Charlson comorbidities index; OR, odds ratio; CI, confidence interval; uACR, urine albumin/creatinine ratio; eGFR, estimated glomerular filtration rate; Cr, creatinine; CysC, cystatin C

**Supplemental Table 5. Subgroup analysis according to age to identify conditions related to discrepant trends of eGFR_Cr_ and eGFR_CysC_**

| **Variables** | **Age ≤ 54 years** | | | | **Age > 54 years** | | | | | |
| --- | --- | --- | --- | --- | --- | --- | --- | --- | --- | --- |
|  | **Model 1** | | **Model 2** | | **Model 1** | | | **Model 2** | | |
|  | **OR (95% CI)** | ***P*** | **OR (95% CI)** | ***P*** | **OR (95% CI)** | ***P*** | **OR (95% CI)** | | ***P*** |  |
| Male | 0.89 (0.29-2.69) | 0.839 |  |  | 6.40 (0.91-50.47) | 0.062 |  | |  |  |
| Body surface area | 0.87 (0.05-12.52) | 0.923 |  |  | 111.95 (0.69-26764.61) | 0.069 |  | |  |  |
| Diastolic blood pressure | 1.00 (0.96-1.03) | 0.857 |  |  | 1.02 (0.96-1.09) | 0.563 |  | |  |  |
| Hemoglobin | 0.85 (0.66-1.11) | 0.237 |  |  | 0.99 (0.62-1.58) | 0.972 |  | |  |  |
| Blood urea nitrogen | 1.02 (0.95-1.08) | 0.568 |  |  | 0.92 (0.75-1.05) | 0.245 | 0.84 (0.81-0.95) | | 0.002 |  |
| Uric acid | 0.91 (0.72-1.15) | 0.439 |  |  | 1.07 (0.67-1.73) | 0.788 |  | |  |  |
| Phosphorus | 0.80 (0.37-1.72) | 0.575 |  |  | 0.50 (0.11-2.10) | 0.342 |  | |  |  |
| Total bilirubin | 1.37 (0.33-5.38) | 0.661 |  |  | 1.47 (0.09-20.77) | 0.774 |  | |  |  |
| uPCR(g/g) |  |  |  |  |  |  |  | |  |  |
| <0.3 | Reference |  |  |  | Reference |  | Reference | |  |  |
| 0.3–0.9 | 1.56 (0.43-5.11) | 0.480 |  |  | 0.91 (0.01-18.06) | 0.954 | 1.75 (0.14-21.97) | | 0.634 |  |
| 1.0–3.0 | 2.99 (0.58-15.41) | 0.189 |  |  | 4.59 (0.02-189.67) | 0.469 | 9.58 (1.47-103.86) | | 0.018 |  |
| ≥3 | 6.53 (0.94-47.57) | 0.072 |  |  | 28.77 (0.14-1398.81) | 0.160 | 56.77 (0.10-649.89) | | 0.000 |  |
| uACR (mg/g) |  |  |  |  |  |  |  | |  |  |
| <30 | Reference |  |  |  | Reference |  |  | |  |  |
| 30–299 | 2.55 (0.82-10.98) | 0.150 |  |  | 4.17 (0.21-625.98) | 0.353 |  | |  |  |
| ≥300 | 1.56 (0.27-10.72) | 0.628 |  |  | 5.43 (0.08-2399.26) | 0.454 |  | |  |  |
| eGFR_CrCysC_ | 1.07 (1.04-1.11) | 0.000 | 1.06 (1.04-1.09) | <0.001 | 1.07 (1.00-1.16) | 0.061 |  | |  |  |

Model 1: Adjusted for sex, body surface area, diastolic blood pressure, hemoglobin, blood urea nitrogen, uric acid, phosphorus, total bilirubin and eGFR_CrCys_.

Model 2: Model 1 with backward elimination method.

OR, odds ratio; CI, confidence interval; uPCR, urine protein/creatinine ratio; uACR, urine albumin/creatinine ratio; eGFR, estimated glomerular filtration rate; Cr, creatinine; CysC, cystatin C

**Supplemental Table 6. Baseline characteristics of study participants for whom eGFR was measured ≥4 times**

| Variables | Total (n=1,451) |
| --- | --- |
| Age (years) | 53.2±12.0 |
| Male (%) | 59.5 |
| Age-adjusted Charlson comorbidity index (%) | 4.0 ±1.8 |
| Low (≤ 3) | 57.1 |
| Moderate (4–5) | 28.0 |
| High (6–7) | 12.8 |
| Very high (≥8) | 2.0 |
| Diabetes mellitus (%) | 29.3 |
| Hypertension (%) | 95.9 |
| Systolic blood pressure (mmHg) | 126.4±14.7 |
| Diastolic blood pressure (mmHg) | 76.7±10.6 |
| Body mass index (kg/m^2^) | 24.5±3.4 |
| Body surface area (m^2^) | 1.7±0.2 |
| Systolic blood pressure (mmHg) | 126.4±14.7 |
| Diastolic blood pressure (mmHg) | 76.7±10.6 |
| Cause of chronic kidney disease (%) |  |
| Diabetic nephropathy | 17.4 |
| Non-diabetic nephropathy | 82.6 |
| eGFR (ml/min/1.73 m^2^) |  |
| eGFR_Cr_ | 57.5±29.6 |
| eGFR_CysC_ | 57.1±31.4 |
| eGFR_CrCysC_ | 56.9±31.1 |
| Laboratory findings |  |
| Hemoglobin (g/dL) | 13.1±1.9 |
| Blood urea nitrogen (mg/dL) |  |
| Uric acid (mg/dL) | 6.9±1.9 |
| Phosphorus (mg/dL) |  |
| Total bilirubin (mg/dL) |  |
| Albumin (g/dL) | 4.2±0.4 |
| Urine protein/creatinine ratio (g/g) | 0.4 (0.1–1.1) |
| <0.3 (%) | 43.5 |
| 0.3–0.9 (%) | 30.1 |
| 1.0–3.0 (%) | 20.1 |
| ≥3 (%) | 6.3 |
| Urine albumin/creatinine ratio (mg/g) | 281.5 (52.8–765.9) |
| <30 (%) | 18.2 |
| 30–299 (%) | 33.1 |
| ≥300 (%) | 48.7 |

eGFR, estimated glomerular filtration rate; Cr, creatinine; CysC, cystatin C.

**Supplemental Table 7. Sensitivity analysis of patients for whom eGFR was measured ≥4 times to identify conditions related to discrepancy between the trends of eGFR_Cr_ and eGFR_CysC_**

|  | Model 1 | | Model 2 | |
| --- | --- | --- | --- | --- |
| Variables | OR (95% CI) | *P* | OR (95% CI) | *P* |
| Age | 0.95 (0.91–0.98) | 0.006 | 0.94 (0.91–0.97) | <0.001 |
| Male | 2.51 (0.90–7.15) | 0.077 | 3.88 (1.53–10.08) | 0.004 |
| Age-adjusted CCI |  |  |  |  |
| Low (≤ 3) | Reference |  |  |  |
| Moderate (4–5) | 1.07 (0.43–2.60) | 0.880 |  |  |
| High (6–7) | 1.13 (0.19–5.01) | 0.879 |  |  |
| Very high (≥8) | 2.49 (0.02–27.95) | 0.596 |  |  |
| Body surface area | 12.51 (1.02–153.08) | 0.048 | 13.48 (1.14–158.69) | 0.039 |
| Diastolic blood pressure | 1.01 (0.98–1.04) | 0.545 |  |  |
| Hemoglobin | 0.85 (0.67–1.07) | 0.174 |  |  |
| Blood urea nitrogen | 0.99 (0.94–1.05) | 0.848 |  |  |
| Uric acid | 0.89 (0.70–1.13) | 0.342 |  |  |
| Phosphorus | 0.92 (0.45–1.87) | 0.823 |  |  |
| Total bilirubin | 1.80 (0.42–7.13) | 0.417 |  |  |
| uPCR (g/g) |  |  |  |  |
| <0.3 | Reference |  | Reference |  |
| 0.3–0.9 | 1.36 (0.31–4.92) | 0.664 | 1.63 (0.65–4.14) | 0.298 |
| 1.0–3.0 | 3.81 (0.66–20.08) | 0.129 | 4.01 (1.65–10.20) | 0.002 |
| ≥3.0 | 11.45 (1.69–72.75) | 0.014 | 12.39 (3.92–39.35) | <0.001 |
| uACR (mg/g) |  |  |  |  |
| <30 | Reference |  |  |  |
| 30–299 | 4.66 (0.96–45.96) | 0.057 |  |  |
| 300 | 3.82 (0.47–52.83) | 0.220 |  |  |
| eGFR_CrCysC_ | 1.07 (1.04–1.11) | <0.001 | 1.07 (1.05–1.10) | <0.001 |

Model 1: Adjusted for age, sex, eGFR_CrCys_ and the variables which had *P* value less than 0.1 in univariate analysis.

Model 2: Model 1 with backward elimination method.

OR, odds ratio; CI, confidence interval; CCI, Charlson comorbidities index; uPCR, urine protein/creatinine ratio; uACR, urine albumin/creatinine ratio; eGFR, estimated glomerular filtration rate; Cr, creatinine; CysC, cystatin C.

**Supplemental Figure 1. Bland-Altman plot between baseline eGFR_Cr_ and eGFR_CysC_**

*Abbreviations*: eGFR, estimated glomerular filtration rate; SD, standard deviation


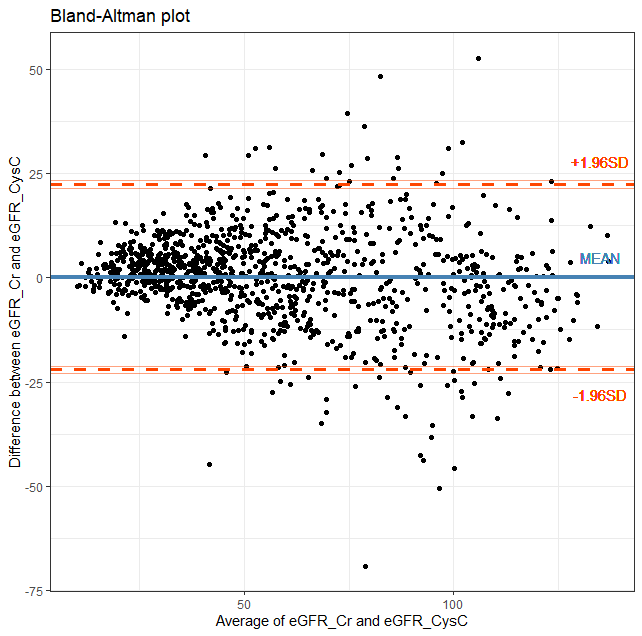


**Supplemental Figure 2. Trajectory patterns of eGFR_Cr_ and eGFR_CysC_ in patients for whom eGFR was measured ≥4 times**


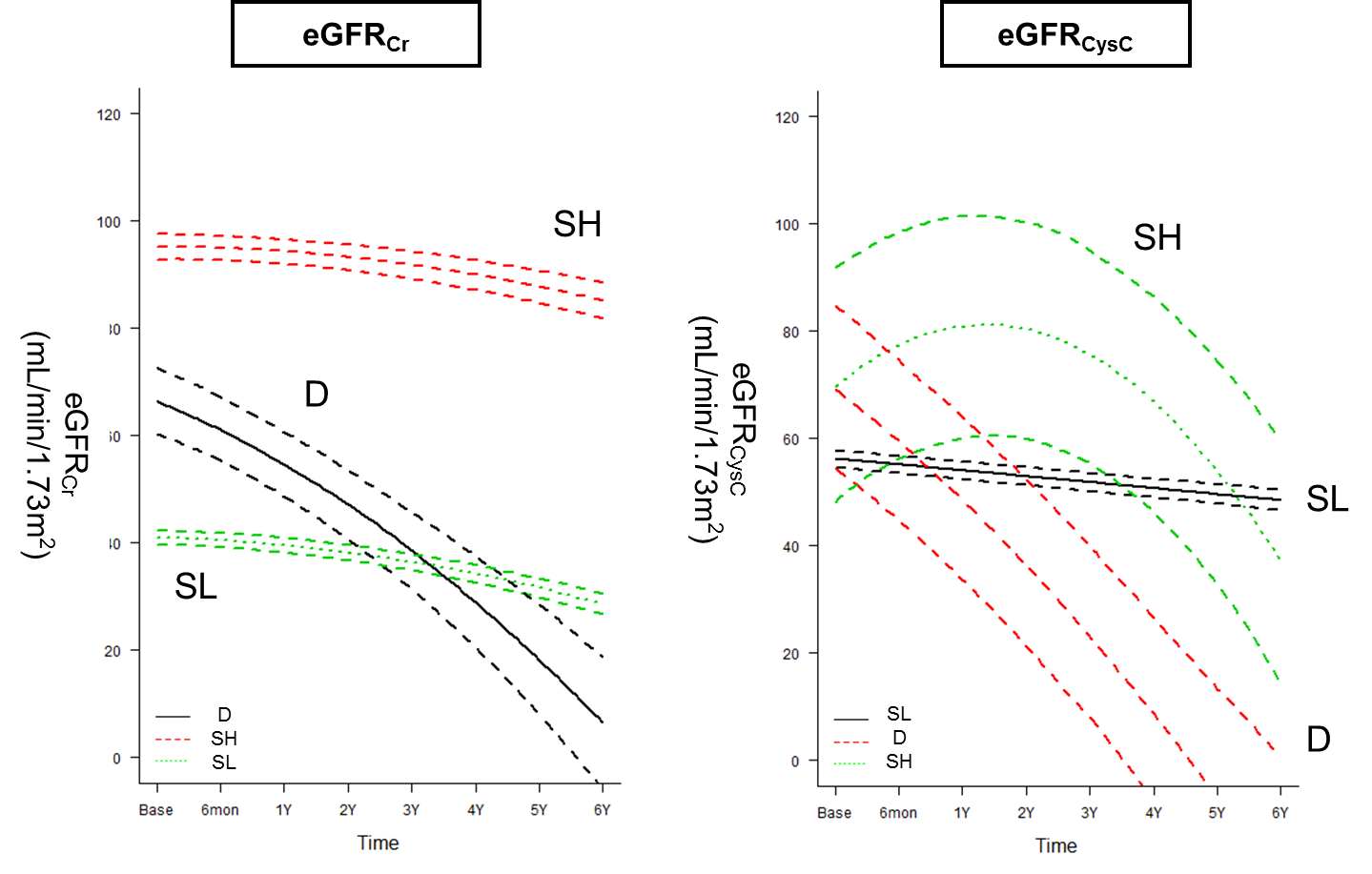


**Supplemental Figure 3. Cross-table between trends of eGFR_Cr_ and eGFR_CysC_ in patients for whom eGFR was measured ≥4 times**


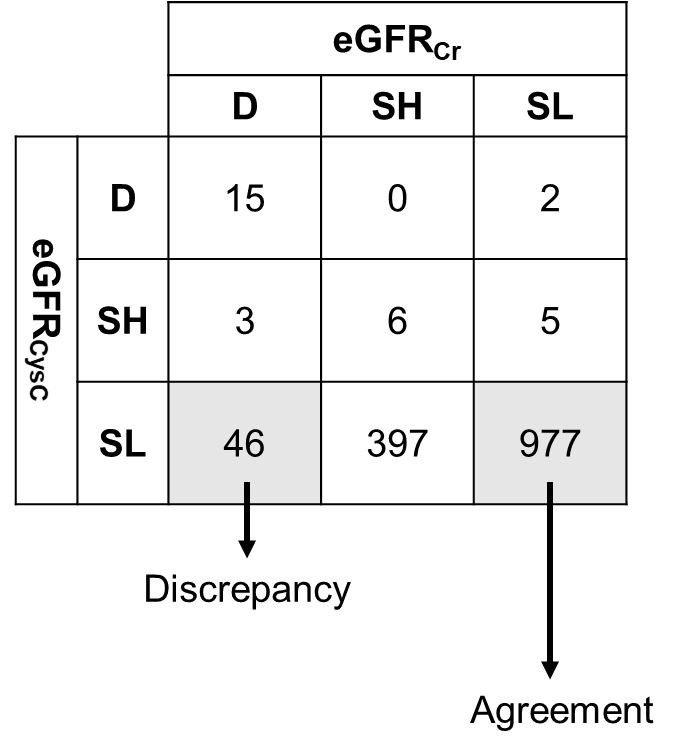

Supplement: Supplementary file 1 — Additional file 1. [file 12882_2020_1932_MOESM1_ESM.docx]
